# Welcome to *Diagnostics*: a New Open Access Journal for the Fast-Growing Field of Medical Diagnostics

**DOI:** 10.3390/diagnostics1010036

**Published:** 2011-11-23

**Authors:** Andreas Kjaer

**Affiliations:** *Editor-in-Chief of Diagnostics*, Department of Clinical Physiology, Nuclear Medicine & PET, National University Hospital, Rigshospitalet, University of Copenhagen, Blegdamsvej 9, DK-2100 Copenhagen, Denmark; E-Mail: akjaer@sund.ku.dk; Tel.: +45-3545-4216.

Diagnostic methods in medicine are currently rapidly evolving and constantly improving. This is true for areas such as molecular diagnostics, biomarkers, as well as medical imaging. As one example, positron emission tomography (PET) has been called the fastest growing medical technology ever. Also, molecular diagnostics, at both the gene and protein levels, are developing rapidly due to advances in technology and, thereby, creating new possibilities. Early and valid diagnosis is crucial for proper treatment of patients. Moreover, advanced diagnostic methods are crucial for the upcoming era of tailored therapy. From an economic viewpoint, the cost of advanced treatments are increasingly indicating the need for better stratification and therapy monitoring of treatment, in order that these advanced treatments are limited to patients able to respond favorably. Collectively, the exciting area of medical diagnostics seems never to have been more important for patients and society.

With the launch of the new journal *Diagnostics*, it is our ambition to give researchers the possibility to publish and read about these new improvements and use of diagnostic methods. With the pace of research within our field, it is essential to have a fast method for dissemination of data available to the research and clinical community. With an open access, peer-review journal, such as *Diagnostics*, we believe to have created exactly that kind of media and I find it a privilege to be founding Editor-in-Chief of this endeavor. There are no restrictions as to the length of papers allowing for great detail in the methodology, which is important if the methods are to be adopted by peers. In addition to regular full-length articles, we encourage authors to also submit reviews and short communications.

The scope of the journal is broad and, intentionally, multidisciplinary. This is based on the increasing use of diagnostic decision algorithms that combine several diagnostic approaches, in order to be more accurate and to be cost-effective. One example could be whether the use of a comparatively cheap biomarker in a blood sample may be used as a gate-keeper to select patients that need a more expensive medical imaging modality. Accordingly, we find it logical to cover all aspects of medical diagnostics in the same journal and thereby support a cross-disciplinary approach and viewpoint. Our editorial board naturally reflects this concept by covering all aspects of medical diagnosis. They have been selected among top-level researchers within various areas of diagnostics, considering both modalities as well as disease entities studied. We, the editorial board and team, are highly committed to the success of the journal and hope that it will grow and flourish. However, the key to being successful is the quality of manuscripts received. Therefore, we encourage all researchers to send in innovative contributions of high quality. We, on our side, can promise a fast handling and review procedure in order to ensure your research is published as quickly as possible.

